# Initial clinical experience with a novel vertebral augmentation system for treatment of symptomatic vertebral compression fractures: A case series of 26 consecutive patients

**DOI:** 10.1186/1471-2474-12-206

**Published:** 2011-09-22

**Authors:** Panagiotis Korovessis, Thomas Repantis, Larry E Miller, Jon E Block

**Affiliations:** 1 Orthopaedic Department, General Hospital "Agios Andreas", 1 Tsertidou str, 26224 Patras, Greece; 2 Miller Scientific Consulting, Inc., 422 Mountain Wasp Drive, Biltmore Lake, NC 28715 USA; 3 Jon E. Block, PhD, Inc., 2210 Jackson Street, Suite 401, San Francisco, CA 94115 USA

**Keywords:** Fracture, Minimally invasive, Osteoporosis, Vertebral augmentation

## Abstract

**Background:**

Minimally invasive vertebral augmentation procedures are widely used to treat vertebral compression fractures although procedural polymethylmethacrylate cement leakage remains common. We report herein our initial experience with a novel vertebral augmentation technique designed to treat symptomatic vertebral osteoporotic fractures and osteolytic metastases with minimal cement extravasation.

**Methods:**

Forty-two vertebral fractures were identified in 26 consecutive patients (mean age 74 ± 9 years). All patients were treated with a novel percutaneous vertebral augmentation device (Kiva^® ^VCF Treatment System, Benvenue Medical, Santa Clara, CA, USA). Indications for surgery included recent (≤ 3 months) symptomatic osteoporotic vertebral fracture (n = 34) and pathologic vertebral fractures (e.g. metabolic bone disease, myeloma, metastasis) (n = 8) located between T10 and S1. Patient outcomes were evaluated pre-treatment and at 2- and 6-month follow-up visits. Postoperative cement extravasation was assessed with computed tomography. Patient-reported back pain was quantified using an 11-point numeric scale. Back-specific functional disability was self-reported with the Oswestry Disability Index on a 0 to 100% scale.

**Results:**

No cases of intraoperative hypotension, respiratory disturbance, neurological deterioration, infection, or death were observed. There were 2 (4.8%) levels where anterior cement leakage was visible radiographically in patients with osteolyses. No intracanal leakage was observed. Back pain scores improved 71% (p < 0.001) from pre-treatment to the 6-month follow-up. Back function improved 56% from baseline to 6 months (p < 0.001).

**Conclusions:**

The initial clinical experience with the Kiva^® ^System demonstrated significant improvements in back pain and function with minimal and clinically insignificant procedural cement leakage.

## Background

Vertebral compression fractures are common injuries with an incidence of 1.4 million each year [1]. Manifestations of fractured vertebrae include severe chronic back pain, disability, and reductions in quality of life [2-7] as well as greater risk for future vertebral fractures [8]. Acute, symptomatic vertebral compression fractures are initially treated with conservative care, which may include bed rest, bracing, analgesic medication, and/or physical rehabilitation and exercise programs. However, vertebral deformity and back pain often persist despite these measures and, therefore, surgery may eventually be required [9]. Minimally invasive vertebral augmentation procedures have been widely used to treat vertebral compression fractures caused by osteoporosis and, less commonly, osteolytic tumors [10-16]. Although the results of these trials are encouraging, procedural polymethylmethacrylate (PMMA) cement leakage remains common with a frequency of 7% to 72% per treated level [10,17-20]. We report herein our initial experience with a novel vertebral augmentation system designed to treat painful vertebral osteoporotic fractures and osteolytic metastases with minimal cement extravasation.

## Methods

### Patients

This single-arm, feasibility trial was conducted at the first author's institution between January 2010 and April 2010. All study procedures were conducted in accordance with the ethical principles stated in the Declaration of Helsinki and this research was approved by the General Hospital "Agios Andreas" (Patras, Greece). Forty-two vertebral augmentation procedures were performed in 26 consecutive patients (mean age 74 ± 9 years; range: 58 to 86 years). Indications for surgery included recent (≤ 3 months) symptomatic osteoporotic vertebral fracture (n = 34) and pathologic vertebral fractures (e.g. metabolic bone disease, myeloma, metastasis) (n = 8) located between T10 and S1. Symptomatic levels were confirmed with x-ray, computed tomography, and/or magnetic resonance imaging.

### Interventions

**Figure 1:**
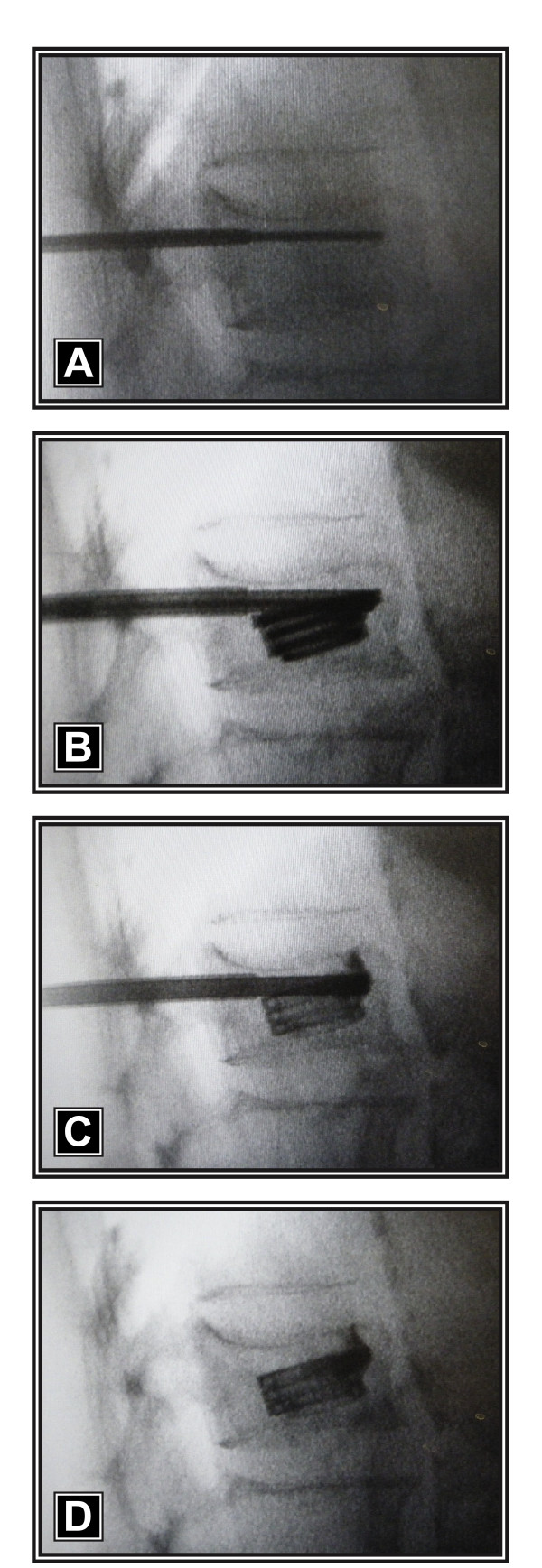
**Intraoperative fluoroscopic images of the Kiva^® ^VCF Treatment System consisting of a percutaneously introduced nitinol coil guidewire advanced through a deployment cannula (a) and then fully coiled within the cancellous portion of the fractured vertebral body (b)**. A radiopaque PEEK Implant is delivered incrementally over the removable guidewire (c) in a continuous loop to form a nesting, cylindrical column providing vertical displacement that results in endplate re-elevation and fracture reduction (d).

**Figure 2:**
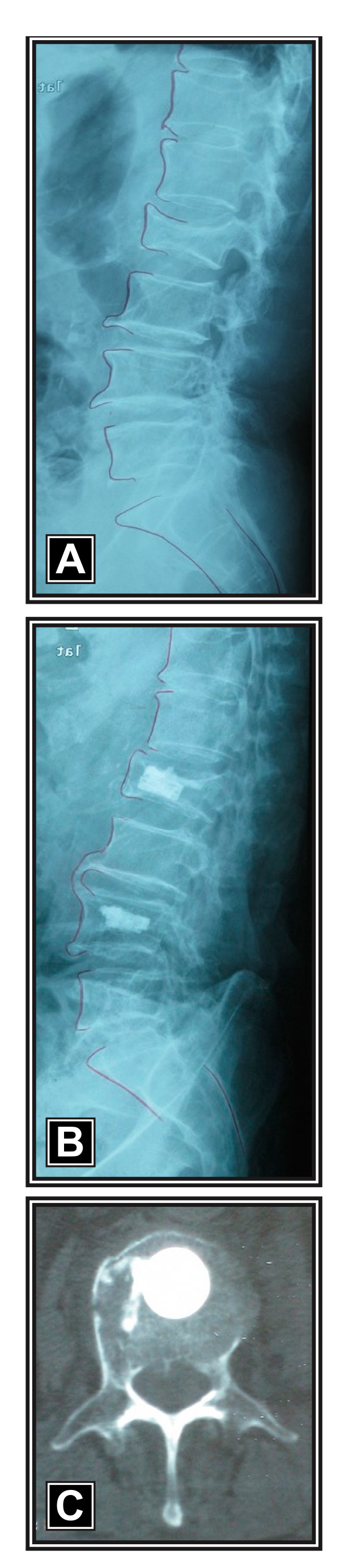
**Preoperative lateral radiograph showing osteoporotic vertebral compression fractures at L2 and L4 in a 72-year-old female **(a). Post-operative radiograph 6 months after treatment with the Kiva^® ^VCF Treatment System demonstrating excellent vertebral fracture reduction (b). Corresponding axial computed tomography scan at 6 months illustrating excellent cement containment within the implant at L2 (c).

The procedures were performed with the patient under general anesthesia and placed in the prone position on an AcroMed frame (DePuy Spine, Inc., Raynham, MA, USA). All patients were treated with a novel percutaneous vertebral augmentation device (Kiva^® ^VCF Treatment System, Benvenue Medical, Santa Clara, CA, USA), which received CE Mark approval in December 2008. The Kiva^® ^System is a sterile, single-use device in which an external delivery handle is used to deploy the Kiva^® ^implant over a nitinol coil guidewire. The coil is first advanced through the deployment cannula (Figure 1a) and into the cancellous portion of the vertebral body (Figure 1b) using an external handle. The Kiva^® ^implant, which is comprised of PEEK-OPTIMA^® ^(Invibio Inc., West Conshohocken, PA, USA) and loaded with 15% barium sulfate to enhance visibility under fluoroscopy, is incrementally advanced over the coil (Figure 1c) to form a nesting, cylindrical column with an *in situ *outer diameter of 20 mm. Up to four loops of the implant may be inserted into the vertebral body for a maximum coil stack height of 12 mm, which re-elevates the endplate, thereby providing the desired vertebral fracture reduction (Figure 1d). After the coil is retracted, radiopaque PMMA cement (2.5-4 cc per level) is injected through the lumen of the implant, thereby interlocking the implant to the vertebral body cancellous bone (Figures 2a and 2b). Percutaneous radiofrequency was applied immediately before implant deployment in patients with metastasis and myeloma.

### Outcomes

Patient outcomes were evaluated pre-treatment and at the 2- and 6-month follow-up visits. Patient-reported back pain was quantified using an 11-point (0 to 10) numeric scale. Back-specific functional disability was self-reported with the Oswestry Disability Index (ODI) (version 2) on a 0 to 100% scale [21]. Postoperative cement extravasation and device-related adverse events were assessed with computed tomography and plain radiographs.

### Data Analysis

Data were analyzed using Predictive Analytics Software (v. 18, SPSS, Inc., Chicago, IL). Continuous data were reported as mean ± standard deviation and categorical data were reported as frequencies and percentages. Longitudinal changes in patient outcomes were analyzed with repeated measures analysis of variance.

## Results

**Figure 3:**
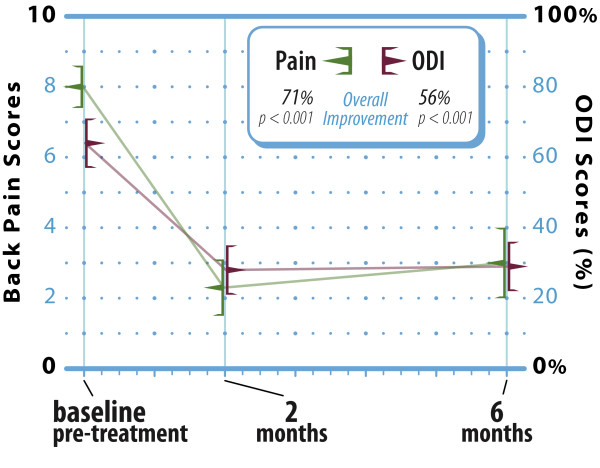
**Improvement in back pain and function through 6 months following vertebral augmentation**. Values are mean ± 95% confidence intervals. ODI: Oswestry Disability Index.

No cases of intraoperative hypotension, respiratory disturbance, neurological deterioration, infection, or death were observed and no blood transfusions were required. There were 2 (4.8%) levels where anterior cement leakage was visible radiographically in patients with osteolyses. No intracanal leakage was observed. No cases of implant migration, subsidence, or refracture at the treated or adjacent levels were reported. Back pain scores improved from 8.0 ± 1.6 at pre-treatment to 2.3 ± 1.0 at 2 months and 3.0 ± 1.5 at 6 months, representing a 71% (p < 0.001) overall improvement. Back function similarly improved from 64 ± 19% at pre-treatment to 28 ± 17% at 2 months and 29 ± 19% at 6 months, representing a 56% (p < 0.001) overall improvement (Figure 3).

## Discussion

The Kiva^® ^System is a novel, safe technique for percutaneous vertebral body augmentation in patients with symptomatic osteoporotic fractures and osteolytic metastases. In our initial experience with this device, we demonstrated improvement in back pain and function through 6 months post-treatment with no significant procedural cement extravasation.

The results of this case series are encouraging. The mean reduction in post-operative pain by 6 months was approximately 5.0 units. These findings compare favorably with the findings from four separate meta-analyses of published studies of the clinical effectiveness of balloon kyphoplasty. Specifically, the mean reductions reported in these meta-analyses for post-operative pain severity scores were 5.1 units from Bouza et al. [10], 5.6 units from Gill et al. [22], 5.4 units from Taylor et al. [19], and 4.6 units from Eck et al. [20].

The effectiveness of vertebral augmentation remains controversial especially since vertebroplasty was reported to have only modest clinical benefit versus sham in two randomized controlled trials [23,24]. These studies enrolled patients with vertebral fracture ages of less than 1 year. However, recent trials of vertebral augmentation that enrolled patients with more acute fractures (6 weeks to 3 months) have reported positive results [17,18,25]. Therefore, the application of vertebral augmentation techniques may be most beneficial when applied soon after fracture, a concept that is in agreement with the initial findings from the current series.

In kyphoplasty and vertebroplasty procedures, PMMA cement may leak laterally to the soft tissues, superiorly or inferiorly into the adjacent disc space, or posteriorly, where it may involve the exiting nerve root or the spinal canal [26]. The Kiva^® ^System, on the other hand, was designed to reduce and stabilize osteoporotic vertebral fractures by deploying a coiled PEEK implant which is then augmented with cement. This technique allows directional cement delivery, which helps to facilitate cement containment. Our first experience with 42 implants confirmed that the Kiva^® ^System was able to control cement leakage with only 2 (7.7%) observed cases, none of which resulted in clinical sequelae. These results compare favorably to cement leakage rates of 7 to 72% reported with kyphoplasty and vertebroplasty [10,17-20].

Limitations of this feasibility study include lack of a control group, a relatively short follow-up period, and lack of objective measures of vertebral height restoration. Despite these limitations, the initial clinical results of this trial are promising and warrant further study in larger series with longer follow-up periods.

## Conclusions

The results from our initial clinical experience with the Kiva^® ^VCF Treatment System demonstrated significant improvements in back pain and function with minimal and clinically insignificant procedural cement leakage.

## Competing interests

LEM and JEB received financial support from Benvenue Medical (Santa Clara, CA, USA) for manuscript development.

## Authors' contributions

PK and TR designed and conducted the study. LEM performed the statistical analyses. LEM and JEB participated in data interpretation. All authors were involved in drafting and critically revising the manuscript. All authors read and approved the final manuscript.

## Pre-publication history

The pre-publication history for this paper can be accessed here:

http://www.biomedcentral.com/1471-2474/12/206/prepub
